# Dr. Wood, on Confluent Small-Pox

**Published:** 1806-07

**Authors:** J. Wood

**Affiliations:** Newcastle


### *To the Editors of the Medical and Physical Journal.*

Gentlemen,

I Mentioned to you in my last communication of a case of confluent small-pox treated by purgatives, nitrate of potash, and the nitric acid; that my original view in selecting that case was for a *general* purpose, as well as the *particular* one of recommending a successful mode of cure in that disease; and that I would probably offer you, for the following number of your Journal, some explanation of the *general object* I had in contemplation. This, I find, I shall still have to defer until another month, that I may now *add* some remarks to my case of confluent small-pox, and that I may also conclude what I have to say at present on the subject of herpes during vaccination, in reply to Mr. Creaser's remarks in your Journal for April, on some parts of my report on vaccination, which appeared in your number for February last. I regret any intervening circumstance that obliges me to postpone my intention, the more, from having seen the notice Doctor Kinglake has taken of Dr. Kentish's paper on the treatment of of burns and scalds, as my observations would include that subject, and probably afford some principles that would explain the difference of opinion between those gentlemen.

To the *first* of my *two present* subjects then, the mode of cure in confluent small-pox, I have to add, that since the date of the case I sent you, viz. January, 1799,* I have always adopted the same treatment detailed in it, in every subsequent case that came under my care, with advantage in proportion *to the degree* I was enabled to enforce it, and to the duration of the disease at the time of my attendance being required. You will recollect, that, in my plan of cure, I do not admit, *in the first instance,* any line to be drawn between confluent and distinct small-pox, yet according to my general principles it *may be* necessary to draw a line between the *different states* occurring in the *same* disease, and at such a line, to change the mode of cure; such however in small-pox I have not hitherto ever found necessary, and since I sent you the case in your last number, a book has fallen into my hands from the library of a physician lately deceased here, which strongly confirms the success of the practice I have pursued; it is entitled, "Johannis Freind, M. D. de purgantibus, in secunda Variolarum Confluentium Febre, adhibendis Epistola." Though the name of Friend can be no stranger to the ear of a medical man, yet I had not seen this little work before; it was printed by William and John Innys, St. Paul's Church Yard, London, anno MDCCXIX, now ninety years ago or nearly, and is an instance, I believe, how in medicine, the best precepts and practice may be forgotten or unknown by the great majority of the medical world. This Treatise was written about ten years before inoculation for the small-pox was introduced, and though it shews strongly the advantages of purgatives and also of occasional blood-letting in that disease, yet it does not mention those also derived from cool air, diet, &c. which were first pointed out, I believe, by the Suttons, and which rendered their practice in inoculation so successful.

Dr.

---

* Some explanation may be necessary, why I allowed so many years to pass without bringing forward this case, if I considered it an important one; but it should be recollected that the cow-pox was introduced towards the end of the same year, and the inoculation of it took place here soon after, when the small-pox disappeared in this neighbourhood, and no case of it occurred for nearly four years, until about October, 1804, since which time it has indeed committed great ravages.

Dr. Freind addresses his Epistola to Dr. Mead, and as many of your readers may not have seen it, I will transcribe one paragraph, which will, I think, be read with pleasure. After having given the history of ten cases, in all of which purgatives produced most striking good effects, he then proceeds:

"Vides, quàm in simili variolarum genere dispar sit morbi ipsius cursus, ratioque affectuum multiplex, et plane dissimillima: vides ægrotos sexûs utriusque, omni tempestatum genere, omnis ætatis, omnis habitûs, omni anni tempore, eodem malo laborantes; quos tamen omnes hæc potissimum *purgandi* via periculo vitæ maximo liberavit. Vides, ut aliis inopinatam præsentissimamque operam attulerit purgatio; ut aliis paulisper subvenerit, cunctantius per vices repetita; ut universis, seriùs aut citiùs, mirabilem in modum saluti fuerit. Nonne enim omnes hi ex ipsis prope lethi angustiis, hoc medicinæ auxilio, emersere? quidam utique, uno quasi ictu, simul et semel erepti; plurimi minutatim, lenteque admodum extricati; ita ut, si quis horum omnium salutem purgationi minime ascribi oportere censeat, idem pari per me licet jure contendat, neque artem medicam unquam exstitisse, neque ullam unquam profuisse cuiquam medicinam. Hæc tamen nota satis atque illustria, non ex otiosorum hominum cerebro effusa, sed ex ipsa, artis magistrà, experientia, eaque multiplici, deducta; non in angulis, non in gurgustiis, inter nutrices anniculas clanculum conficta, sed in luce atque conspectu hominum, maximeque intra nobilium parietes, coram testibus idoneis gesta atque probata."

I have given the more importance to these remarks, and to the general plan of cure I have given you as most successful in small-pox, not only because they exhibit a practice very opposite to one that was much inculcated here, and is yet adhered to by some, viz. that of bark, wine, &c. but because also, however sanguine I was, at one time, as to think that the cow-pox would completely exterminate the small-pox, I must now candidly confess, I do not expect that such unanimity of opinion amongst mankind with respect to cow-pox inoculation will ever so far prevail as to leave no objects exposed to the ravages of small-pox, not to mention how many besides will always be left liable to its attack from ignorance and neglect.

I will now, gentlemen, beg leave to turn my attention to my *second* proposed subject of my *present* paper, viz. Mr. Creaser's remarks in your number for April, on some parts parts of my late report on vaccination. I must first observe, that Mr. C. does not attend to the circumstance that the opinion I gave with regard to herpes not affecting the security obtained by the cow-pox against the influence of the small-pox, *was an answer,* and not *advanced,* being compelled either to give this opinion, or to acknowledge that my report was defective in "sterling instruction as well as observation," as Mr. Clement had thought proper to say. Had it not been thus, I probably would have waited for the farther evidence of fact, before I advanced an opinion contrary to the observations of others. Still I do not regret it; it has led, as I certainly had reason to expect, to some discussion, which always does good, when conducted at once with freedom, liberality, and becoming respect. Even Mr. Creaser has not attended in his remarks on my report, to that liberality which I conceive requisite, in suspecting, or rather insinuating, that my opinion was influenced by *party* feelings; and had it not been on this account his remarks would not have called for any particular answer from me, as all that could have been said would have been, that Mr. C. had observed the presence of herpes to affect the vaccine pustule, and the consequent security against small-pox, and that it had not been observed to do so here, farther observations therefore would be necessary to ascertain the circumstance, which seems at present to stand thus. It has been observed at the Dispensary here, that the presence of herpes does not affect the vaccine pustule, nor the consequent security. It has been thought by the Somerset Jennerian Society that it does. It has been observed by the Vaccine Institution in Dublin, that herpetic and other eruptions produce difficulty in communicating cow-pox, and occasional deviations in its progress; and against this we have the statement in answer from the Original Vaccine Pock Institution in London, that herpetic or other eruptions have rarely occasioned any difficulty in communicating the infection, or have varied the progress of vaccination, except from the patients in such cases scratching or rubbing their arms; and that neither scrofula, rickets, nor other chronic complaints alter the course of the vaccine affection; such also are the sentiments of Mr. Ring, and such are also precisely our observations at the Dispensary here, which I had no sooner brought forward in your Journal, than I saw them supported by those also which I have now quoted. Thus, according to Mr. Creaser's proposal for determining this point, and on which he joins issue with me, the weight at present seems in favour of my opinion; for although on the one side the opinion formed may be said to be from negative circumstances, yet in *true philosophy* I apprehend the conclusion is of a positive nature, as much so, as we would be decided by any experiment in chemistry, which though conducted negatively, yet by the result produces positive proof; effects must follow causes, and if the effect does not take place, we may legitimately conclude, that the cause does not exist; and the opinion is formed. All that Mr. Creaser therefore could say, is, that I had formed such an opinion, because such effects had not taken place here, and that he had formed an opposite opinion, because he saw such effects, which he *attributed* to certain causes. How, therefore, are the interests of philosophical truth so involved by *my* opinion, as Mr. Creaser supposes?

By such a view only can we turn the scale in favour of any opinion, when a contrary one is maintained, and both are deduced from observation and experience; without such a view, facts opposed to facts would only surprise and confound, or one party would be deemed incapable of making just or accurate observations. It was no matter of surprise that such different opinions were formed respecting the influenza being communicated by contagion or not, as each individual might be easily deceived as to the grounds on which he formed his opinion; but on the subject of herpes affecting or not affecting the vaccine pustule, the grounds of opinion are more decisive, and that any difference should exist appears prima facie more strange. We cannot therefore go farther at present than say, with the original vaccine pock institution, in answer to Dr. Clark of Dublin, that herpes *had not* affected the vaccine pustule in their experience, nor, that it *would not*.

We have been so fortunate here as not to meet with any *spurious* cow pock or pustules, except the derangement produced by scratching or rubbing may be called so. I confess, indeed, that I never liked *that term* from the first, because I wished, what I now believe, that the cow-pox *will carry all before it*, and is not impeded in its progress by herpes or any other eruption.

Let me now proceed to reply to Mr. Creaser's insinuation, that the opinion I gave was influenced by feelings of party; by shewing, as I have done, that my opinion was not voluntarily advanced, but that I was compelled to bring it forward at the time I did, I trust I sufficiently do away Mr. Creaser's scepticism, "that I was inclined to be cautious in my demonstration of it, because it militated against that of Dr. Jenner." If, however, Mr. C. should not think so, and to remove any mark of the "character of of party," with which my report impressed Mr. C. I have no objection to say more; truth alone is my object, and if it has appeared contaminated with feelings to which it ought to be superior, I the more readily try to clear it of them; and I think I cannot do this in the opinion of Mr. Creaser, more concisely and effectually than by agreeing with him in his sentiments on the "history of the early proceedings in vaccination," to which he alludes, and which I read at the time in his "Observations, &c." And while I do this, and also agree with him in deprecating the existence of a difference of sentiment, and wishing for unanimity of exertion against the common enemy, yet I must be allowed to say, that Dr. Pearson, by his individual exertions, did raise ramparts of defence in so scientific a manner round the discovery of Dr. Jenner, as to secure the blessing to mankind. Were we to march against the common enemy, it would be necessary to agree on certain principles of union, and to defer the discussion of some points until the enemy was defeated; but the occasion requires only actions of defence, and the cause is so good as not to admit of any fear of its success, while its advocates are employed in the liberal discussion of its particular qualities. On this subject I cannot but express my wish that all the friends of the cow-pox had acted only *on the defensive*, (like Dr. Pearson); its enemies should not have been pursued beyond a certain line; after which, "let them alone, and they will die of themselves."

I hope Mr. Clement's hasty remarks on my first report, will point out the propriety of one not hastily condemning the opinion on silence of another or any part of a subject; and that Mr. Creaser will see that any appearance of ground for the suspicion he entertained arose from Mr. Clement's *partial* views. I hope too that gentlemen, in future, when they express a difference of sentiment on any subject, will do it as if they were unanimous in the investigation of truth, and not desirous of hurting the personal feelings of any individual, which leads to controversy that acts like poison on the mind, while candid investigation not only sits easy on it, but strengthens and invigorates it. With this view, I will conclude the subject with a case of vaccine inoculation during the presence of inveterate herpes, at the infirmary in this town. The person inoculated was a young woman, who had had small-pox, and thus their different points were ascertained by inoculation with cow-pox matter.

1st. That cow-pox will take place after small-pox, which proves that the former is not any modification of the latter.

 2d. That

2d. That herpes of very long standing did not in the least prevent the infection of the cow-pox taking place, nor affect the appearance of the pustule.

And, 3dly, That the constitutional action induced by cow-pox tends to *remove* the herpetic disease. The case will also shew, and I have ascertained the same in numerous instances, that herpes frequently *follows* the small-pox.

Ann Robson, 20 years of age, had when 8 years old the small-pox in the casual way, with a very great eruption; immediately after she was affected with spots on her face, (the herpes pustulosus), which continued getting worse every season for twelve years; the spots had spread and affected her nose for six years, during which time she had tried every means for the cure of so disagreeable a complaint without any good effect, and had almost despaired of its ever being removed. In this situation I saw her, and proposed inoculation for the cow-pox, to which she readily agreed, and she was inoculated on the 3d of March last, in both arms, which put on the usual appearance, the left arm always rather more forward than the right, until the 8th day, when she experienced a little general indisposition, heat in her face, and pain under the arms, which continued four days, during which time her face was more inflamed; the vesicle on the parts inoculated did not appear so *full* nor *tender* as it does in children, and the areola was neither so evident, nor so permanent; it was very perceptible on the morning of the 9th day, but only for a few hours; as the inflammation abated on the arms, so did the heat and redness on her face, and as her arms scabbed, so the herpetic eruption scabbed and dried, and fell off, leaving the greatest part healed underneath; and had any *external** application been at the same time used, there is every reason to think that nearly, if not the whole of the herpetic ulceration would have been healed. She was seen during the inoculation by several professional gentlemen, who were perfectly satisfied with the appearance of her arms, and who observed that the vaccine vesicle in *adults* often differed, in the manner I have described, from that which takes place in *children*. Has the observation been made before? I have prefaced this case with the conclusions I had drawn from it; it will speak so plain for itself, as not to require from me any further comment.

I am, &c.

*Newcastle, May* 27, 1806. J. WOOD.

---

* It was deemed a more fair experiment not to use at the same timeany *external* application, except milk and water for the sake of cleanliness.

